# Preparation and Antitumor Evaluation of Four Pentacyclic Triterpenoids and 10-Hydroxycamptothecin Self-Assembled Nanoparticles

**DOI:** 10.3390/pharmaceutics17121577

**Published:** 2025-12-08

**Authors:** Tingen Zhang, Yiwen Hu, Wenzhuo Yang, Xiaochao Huang, Linhui Zhang, Xiaotong Hou, Pengyu Shen, Ruihong Jian, Zhidong Liu, Jiaxin Pi

**Affiliations:** 1 State Key Laboratory of Chinese Medicine Modernization, Tianjin University of Traditional Chinese Medicine, Tianjin 301617, China; zhangtingen9912@163.com (T.Z.); huyiwen108@163.com (Y.H.); 18502240914@163.com (W.Y.); 18223911503@163.com (X.H.); 15891891712@163.com (L.Z.); h13363815891@163.com (X.H.); shenpengyu0210@163.com (P.S.); jrh2530586919@163.com (R.J.); liuzhidong@tjutcm.edu.cn (Z.L.); 2 Engineering Research Center of Modern Chinese Medicine Discovery and Preparation Technique, Tianjin University of Traditional Chinese Medicine, Ministry of Education, Tianjin 301617, China

**Keywords:** pentacyclic triterpenoids, hydroxycamptothecin, self-assembly, antitumor

## Abstract

**Background/Objectives**: A carrier-free self-assembled nanomedicine delivery system refers to a high drug-loading nanomedicine delivery system prepared by one or more active drug ingredients through supramolecular self-assembly, which has the advantages of high drug-loading and a simple preparation process, enabling multidrug synergistic therapy. 10-hydroxycamptothecin (HCPT) have active antitumor effects. Pentacyclic triterpenes are natural active components with a wide range of pharmacological activities. This study aimed to investigate the impact of structural types on the self-assembly of pentacyclic triterpenes and HCPT. **Methods**: Molecular docking studies were performed. Self-assembled nanoparticles were designed by co-assembling ursolic acid (UA), asiatic acid (AA), oleanic acid (OA), and betulinic acid (BA) with HCPT via anti-solvent precipitation combined with ultrasonication, followed by characterization. Cytotoxicity assays using the CCK-8 method revealed that the prepared self-assembled nanoparticles exhibited concentration-dependent inhibitory effects against A375, AGS, HCT-116, and HepG2 tumor cells. Confocal laser scanning microscopy (CLSM) indicated that UA/HCPT nanoparticles (UA/HCPT-NPs) were more efficiently internalized and accumulated in cells compared with the UA + HCPT physical mixture. **Results**: Both in vitro and in vivo results demonstrated that the self-assembled nanoparticles significantly enhanced antitumor efficacy while exerting minimal toxicity on major organs within the tested dose range. **Conclusions**: In summary, these findings highlight that pentacyclic triterpenoids components possess significant self-assembly potential, and that dual-drug co-delivery via self-assembled nanoparticles represents as a promising strategy for cancer therapy.

## 1. Introduction

The self-assembly phenomenon refers to the process by which internal units of a disordered system spontaneously form an ordered structure without external intervention [1]. This phenomenon is widespread and has significant applications in various fields. For instance, DNA bases can self-assemble into well-defined two-dimensional and three-dimensional nanostructures through specific hydrogen bonding interactions [2]. Additionally, peptide molecules or small organic molecules can self-assemble to form nanofibers or tubular structures, which have promising applications in drug delivery systems [3].

In recent years, the utilization of the supramolecular self-assembly capability of drugs to construct carrier-free self-assembled nanomedicine delivery systems via a “bottom-up” strategy has been widely studied. These systems are formed through the synergistic action of non-covalent interactions, including hydrogen bonding, π-π stacking, hydrophobic interactions, electrostatic interactions, and van der Waals forces. Compared with traditional carrier-based nano formulations, carrier-free nanodrugs offer distinct advantages, such as a simplified preparation process, high drug-loading capacity, and the elimination of potential toxicity and immunogenicity caused by carrier excipients in organisms [4].

Studies have demonstrated that carrier-free self-assembled nanomedicines enable drug co-delivery and combination therapy by co-assembly of multiple drug molecules. Guo et al. [5] employed anti-solvent precipitation to prepare co-assembled nanoparticles of irinotecan hydrochloride and curcumin, which not only improved the bioavailability of curcumin but also utilized curcumin as a photosensitizer under light exposure. This approach enhanced tumor-site targeting and promoted drug accumulation and cellular uptake within tumors. Additionally, the formation of multidrug self-assembled nanosystems can lead to more stable structures, a phenomenon attributed to the equilibrium of intermolecular interaction forces [6]. For instance, paclitaxel (PTX) exhibits strong hydrophobicity but poor self-assembled nanoparticle stability, causing rapid aggregation and precipitation in water, which hinders the preparation of stable self-assembled nanoparticles. However, Jiang et al. [7] introduced indocyanine green (ICG) and PTX through hydrophobic co-assembly to form a stable core–shell structure, significantly enhancing the stability of PTX self-assembly systems. This drug combination was proved to effectively overcome tumor cell multidrug resistance and enhance antitumor efficacy.

Pentacyclic triterpenoids are a class of natural active ingredients widely distributed in Chinese herbal medicines. Based on their glycosidic structural characteristics, these compounds can be categorized into four types: oleanolane-type (e.g., oleanolic acid, glycyrrhizic acid), ursane-type (e.g., ursolic acid, asiatic acid), lupulane-type (e.g., lupulin, betulinic acid), and corkypropane-type (e.g., rasperidinone, corkypropane). These substances exhibit diverse pharmacological activities, including antitumor, anti-inflammatory, antibacterial, and hepatoprotective effects, with antitumor mechanisms being the most intensively studied. Extensive research has demonstrated that numerous pentacyclic triterpenoids possess antitumor activity, exerting their effects through multiple pathways such as inhibiting proliferation, inducing apoptosis, and regulating immunity [8,9]. For example, oleanolic acid (OA) induces cellular autophagy by modulating p62 expression, triggers apoptosis via the mitochondrial pathway, arrests the cell cycle by impeding IL-6/STAT3 signaling activation [10,11,12], and prevents tumorigenesis by blocking carcinogen activation and DNA oxidative damage [13]. Ursolic acid (UA) and betulinic acid (BA) regulate cellular processes (e.g., differentiation, proliferation, apoptosis, and angiogenesis) by inhibiting the STAT3 signaling pathway [14,15]. Asiatic acid (AA) exerts antitumor effects by suppressing tumor cell proliferation, inducing apoptosis, and reversing tumor cell multidrug resistance [16,17,18]. However, the low aqueous solubility, slow dissolution rate, and poor bioavailability of pentacyclic triterpenoids severely limit their clinical therapeutic efficacy. Therefore, the development of more efficient and safe drug delivery systems is urgently required.

10-Hydroxycamptothecine (HCPT), an alkaloid extracted from *Camptotheca acuminata* Decne, has demonstrated inhibitory effects against a variety of tumor cells, including hepatocellular carcinoma and gastric carcinoma [19]. Its primary mechanism of action involves interacting with DNA during the S phase, selectively inhibiting DNA topoisomerase I synthesis to block the degradation of cleaved DNA strands [20]. This mechanism suppresses cell proliferation and exerts antineoplastic effects. Notably, HCPT exhibits no cross-resistance with commonly used clinical antitumor drugs, making it a promising candidate for combination therapy. However, its extremely poor solubility necessitates clinical use of its sodium salt injection. Unfortunately, lactone ring opening reduces drug efficacy, limiting its clinical application. Studies have demonstrated that HCPT can self-assemble into short rod-shaped nanoparticles [21] and co-assemble with other drugs for synergistic delivery [22,23]. For example, HCPT and adriamycin form dual-drug co-assembled nanoparticles by solvent exchange [24], improving drug uptake by MCF-7 breast cancer cells and strengthening antitumor effects.

Pentacyclic triterpenoids share analogous rigid skeletal structures with HCPT, potentially enabling high affinity for two-drug self-assembly. Both molecules contain multiple functional groups (e.g., hydroxyl and carboxyl groups), which facilitate hydrogen bond formation.

In this study, self-assembled nanoparticles were prepared by co-assembling HCPT with four pentacyclic triterpenoids—ursolic acid (UA), asiatic acid (AA), oleanic acid (OA), and betulinic acid (BA)—to investigate the influence of different chemical structures on the two-drug self-assembly process. A series of characterizations and in vitro/in vivo antitumor pharmacodynamics experiments were conducted to determine whether these co-assembled nanoparticles enhance synergistic antitumor effects. This research aims to provide a scientific basis for multidrug co-delivery strategies and expand new horizons for combinatorial antitumor therapy.

## 2. Materials and Methods

### 2.1. Materials

Ursolic acid (purity ≥ 98%), betulinic acid (purity ≥ 98%), and asiatic acid (purity ≥ 95%) were purchased from Shanghai Meryer Chemical Technology Co., Ltd. (Shanghai, China). Oleanic acid (purity ≥ 96%) was obtained from Shanghai Yuanye Biotechnology Co. Ltd. (Shanghai, China). 10-Hydroxycamptothecine (HCPT) (purity ≥ 98%) was purchased from Shanghai Macklin Biochemical Co., Ltd. (Shanghai, China). Dimethyl sulfoxide (DMSO, analytical grade) and Poloxamer 188 (P188) were supplied from Tianjin Damao Technology Co., Ltd. (Tianjin, China) and BASF Corporation (Ludwigshafen, Germany), respectively. HPLC-grade methanol and phosphoric acid were obtained from Thermo Fisher Scientific Inc. (Waltham, MA, USA). Mannitol was purchased from Tianjin Zhonglian Chemical Reagent Co., Ltd. (Tianjin, China). Cell culture media (McCoy’s 5A, DMEM, F-12k, RPMI 1640), penicillin–streptomycin solution (PS), trypsin-EDTA buffer (0.25%), and fetal bovine serum (FBS) was sourced from Gibco (Grand Island, NY, USA). Polyoxyl(35) castor oil and PEG400 were obtained from BASF Corporation (Ludwigshafen, Germany) and Shanghai Yuanye Biotechnology Co. Ltd. (Shanghai, China), respectively. Clinical chemistry kits (CRE, AST, ALT) were obtained from Nanjing Jiancheng Bioengineering Institute (Nanjing, China).

Male BALB/c mice (6–8 weeks old, weighing 18 ± 2 g) were obtained from SPF Biotechnology Co. Ltd. (Beijing, China). Prior to experiments, mice were housed in a standard facility with free access to tap water and food. All animal procedures were approved by the Animal Ethics Committee of Tianjin University of Traditional Chinese Medicine (Approval No.TCM-LAEC2023242z1005).

### 2.2. Molecular Docking of Self-Assembled Nanoparticles

To elucidate the intermolecular interaction between four pentacyclic triterpenes and HCPT, molecular docking studies were performed to evaluate their docking scores. The 2D structures of four pentacyclic triterpenes (ursolic acid, asiatic acid, oleanolic acid, betulinic acid) and HCPT were retrieved from PubChem, converted to 3D models using Chembio 3D (22.0.0, PerkinElmer, Shelton, CT, USA), and saved as MOL2 files for docking. Molecular docking was conducted using AutoDock Vina (1.1.2, The Scripps Research Institute, La Jolla, CA, USA) [25], and the 3D interaction models between pentacyclic triterpenes and HCPT were visualized via Discovery Studio Visualizer (2025, Dassault Systèmes BIOVIA, Waltham, MA, USA).

### 2.3. Preparation of Self-Assembled Nanoparticles

Inspired by previous studies on the supramolecular self-assembly capability of pentacyclic triterpenoids [26,27], the following protocol was developed: UA and HCPT were weighed at a 1:1 molar ratio and dissolved in DMSO to form a 20 mg/mL organic phase. The aqueous phase was set 0.2 mg/mL P188 solution. The organic phase was injected into the aqueous phase at a 1:20 volume ratio at a constant rate of 1 mL/min under magnetic stirring (1200 rpm, 5min). The crude suspension was further dispersed by ultrasonic cell disruption for 8 min (200 W, 2 s on/3 s off cycle), yielding UA/HCPT-NPs.

After optimizing the UA/HCPT-NPs preparation method, AA/HCPT-NPs, OA/HCPT-NPs, and BA/HCPT-NPs were prepared using the same protocol. In addition, lyophilization conditions for UA/HCPT-NPs were optimized, with 3% mannitol identified as the lyophilization protective agent.

### 2.4. Pharmaceutics Evaluation

#### 2.4.1. Appearance, Particle Size and Zeta Potential

The appearance and physical properties of UA/HCPT-NPs, AA/HCPT-NPs, OA/HCPT-NPs, and BA/HCPT-NPs were recorded. Their Tyndall effects were observed and compared with those of crude suspensions of UA, AA, OA, BA, HCPT, and deionized water.

The particle size and zeta potential of the fabricated nanoparticles were measured in triplicate using a Nanozetasizer laser (Malvern Panalytical, Malvern, UK) particle size analyzer.

#### 2.4.2. Stability of Self-Assembled Nanoparticles

Three batches of each nanoparticle formulation (UA/HCPT-NPs, AA/HCPT-NPs, OA/HCPT-NPs, BA/HCPT-NPs) were prepared in parallel, and their stability was observed after being stored at 4 °C and room temperature (RT) for 30 days, respectively.

#### 2.4.3. Surface Morphology

The morphological features of nanoparticles were observed using a scanning electron microscope (Tescan Mira, Shanghai, China). Samples were diluted to appropriate concentrations, spray-coated with gold, and imaged under high vacuum.

#### 2.4.4. Differential Scanning Calorimetric Analysis

Differential Scanning Calorimetry analyses were performed using a DSC 3500 Sirius instrument (NETZSCH, Selb, Germany) to characterize the thermal properties of UA, AA, OA, BA, HCPT, drug mixtures, and self-assembled nanoparticles. Approximately 5 mg of each sample were loaded into a crucible, and measurements were conducted under a nitrogen flow (20 mL/min) with a heating rate of 10 °C/min from 20 °C to 350 °C. A blank crucible served as the reference.

#### 2.4.5. Fourier-Transform Infrared Spectroscopy (FT-IR) Analysis

FT-IR spectra were acquired using an FT-IR spectrophotometer (Nicolet 6700, Madison, WI, USA) in the wavenumber range of 400–4000 cm^−1^ to characterize the properties of UA, AA, OA, BA, HCPT, drug mixtures, and self-assembled nanoparticles.

### 2.5. Antitumor Effects In Vitro

#### 2.5.1. Cell Culture

HCT-116 (colon cancer), HepG2 (hepatocellular carcinoma), A375 (melanoma), and AGS (gastric adenocarcinoma) cells were cultured in McCoy’s 5A, RPMI 1640, DMEM, and F-12k media, respectively, supplemented with 10% FBS and 1% penicillin–streptomycin. 

#### 2.5.2. In Vitro Cytotoxicity

Cell viability was assessed using the CCK-8 assay. Cells were seeded in 96-well plates at a density of 1 × 10^5^ cell/well and incubated until ~80% confluent. Treatment included HCPT, UA, AA, OA, BA, drug mixtures, and nanoparticles at concentrations of 0, 0.1, 0.5, 1, 10, 50, and 100 μM drug-containing complete medium solution (based on HCPT equivalent). After 48 h of incubation, the drug-containing medium was aspirated, and 100 μL of fresh medium containing 10% CCK-8 was added to each well for a 40 min incubation. The absorbance was measured at 450 nm using a microplate reader (Tecan, Maennedorf, Switzerland). Untreated cells in the medium served as the control. The percentages of cell viability were calculated using the following formula:

Cell viability (%) = (A_drug_ − A_blank_)/(A_control_ − A_blank_) × 100%

Half-maximal inhibitory concentration (IC_50_) values were calculated for each dosing group were calculated using nonlinear regression analysis in GraphPad Prism 9.

#### 2.5.3. Cell Uptake

The intrinsic green fluorescence of HCPT (excitation: 390 nm, emission: 435 nm) was used to visualize cellular uptake via confocal laser scanning microscopy (CLSM). HepG2 cells were seeded on coverslips in 12-well plates and treated with UA/HCPT-NPs or UA + HCPT physical mixtures (5 μM HCPT equivalent) for 0, 2, 4, and 6 h. Subsequently, cells were washed with PBS, stained with DiI (4 μM, member marker; excitation: 550 nm, emission: 570 nm) for 30 min, and imaged using a CLSM system (Leica, Wetzlar, Germany).

### 2.6. In Vivo Antitumor Effects

#### 2.6.1. Cell Culture for Tumor Model

H22 cells were maintained in RPMI 1640 medium with 10% FBS and 1% PS and passaged regularly for tumor model establishment.

#### 2.6.2. Animal Model and Treatment

To establish tumor models [28], male BALB/c mice (6–8 weeks old) were subcutaneously inoculated with 0.1mL of H22 cell suspension (1 × 10^6^ cells) into the right axillary region. Tumor growth was monitored daily until the volume reached ~75mm^3^.

Since UA and HCPT are insoluble in water, they were dissolved in a mixture of ethanol/polyoxyl(35) castor oil/PEG400 (2:3:5, *v*/*v*/*v*) for the physical mixture group. For UA, HCPT, dual-pharmacological mixture, and UA/HCPT NPs, add ethanol after sonication, followed by polyoxyethylene 35 castor oil and PEG400, and then continue to dilute with saline to obtain the relative concentration of HCPT 0.5 mg/mL UA solution, 0.5 mg/mL UA + HCPT physical mixture solution, HCPT containing 0.75, 0.5, 0.25 mg/mL UA/HCPT-NPs solution, respectively. The UA solution contained a relative concentration of 0.5 mg/mL of HCPT, 0.5 mg/mL of a physical mixture solution of UA and HCPT, and 0.25 mg/mL of a solution of HCPT-containing UA/HCPT-NPs.

Tumor-bearing mice were randomly divided into seven groups (n = 6): saline, UA (5 mg/kg HCPT equivalent), HCPT (positive control, 5 mg/kg), UA + HCPT physical mixture (5 mg/kg HCPT equivalent), and UA/HCPT-NPs (2.5, 5, and 7.5 mg/kg HCPT equivalent). Treatments were administered via tail vein injection every two days. The body weight and tumor volume of the mice (calculated as length × width^2^/2) were recorded every two days [29]. The sacrifice of mice was achieved by cervical dislocation.

tumor volume (mm^3^) = length (mm) × width^2^ (mm^2^)/2.

#### 2.6.3. Changes in Body Weight

Body weight was recorded before the first tail vein administration and before each subsequent administration. Weight variation was expressed as follows:

Weight percentage (%) = m_0_/m_X_ × 100%

Inhibition Rate of body weight (IRBW, %) = 1 − m_x_/m_y_ × 100%

where m_0_ = initial weight, m_x_ = weight at day x, and m_y_ = weight post-treatment.

#### 2.6.4. Tumor Growth

Tumor dimensions were measured with vernier calipers, and tumor volume was calculated as above. On day 14, mice were sacrificed, and tumors were excised and weighed. Tumor weight inhibition rate (TWIR) and tumor growth inhibition (TGI) was calculated as follows:

TWIR (%) = 1 − m_treament_/m_saline_ × 100%

TGI (%) = [1 − (T_b_ − T_a_)/(C_b_ − C_a_)] × 100%

where T_a_/T_b_ = tumor volume before/after treatment in the test group and C_a_/C_b_ = tumor volume before/after treatment in the saline group.

#### 2.6.5. Immunohistochemistry

Tumor tissues were stained for Ki67 protein (a marker of tumor cell proliferation) using standard immunohistochemical protocols. Stained sections were visualized under a light microscope, and positive cell percentages were quantified.

#### 2.6.6. Organ Coefficients

Organ coefficients (heart, liver, spleen, lung, kidney) were calculated as follows:

Organ Coefficient (%) = w_organ_/w_body_ × 100%

where w_organ_ = organ weight and w_body_ =mouse body weight.

#### 2.6.7. Histopathological Analysis

Major organs were harvested, fixed in 4% (*v*/*v*) paraformaldehyde solution, processed into paraffin sections, and stained with hematoxylin–eosin (HE). Tissue morphology was evaluated under an optical microscope to assess treatment-related toxicity.

#### 2.6.8. Serum Biochemistry

Serum levels of creatinine (Cre), alanine transaminase (ALT), and aspartate aminotransferase (AST) were measured using commercial kits to evaluate hepatic and renal function.

### 2.7. Statistical Analysis

All data calculations and analyses were performed using GraphPad Prism 9. Experimental results are presented as mean ± standard deviation (SD). Statistical comparisons between groups were performed using Student’s *t*-test and one-way analysis of variance (ANOVA). Significance levels were defined as follows: * *p* < 0.05, ** *p* < 0.01, *** *p* < 0.001, **** *p* < 0.0001.

## 3. Results

### 3.1. Molecular Docking

**Figure 1:**
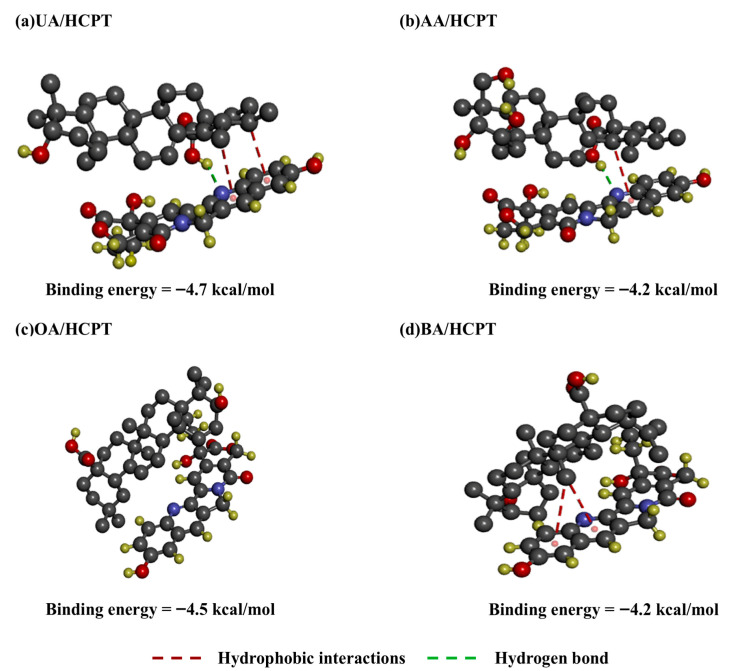
Molecular docking simulation of pentacyclic triterpenoids with HCPT. (**a**) UA/HCPT, (**b**) AA/HCPT, (**c**) OA/HCPT, and (**d**) BA/HCPT molecule complexes with their 3D structural models. Green dashed lines denote hydrogen bonds, and red dashed lines indicate hydrophobic interactions.

Hydrogen bond formation and hydrophobic interactions between four pentacyclic triterpenoids and HCPT were analyzed via AutoDock Vina 1.1.2 and Discovery Studio Client. As shown in Figure 1, UA, AA, OA, and BA successfully docked with HCPT. Pentacyclic triterpenes exhibited a general self-assembly phenomenon primarily driven by the synergistic action of hydrogen bonding and hydrophobic interactions. Specifically, one hydrogen bond and two pairs of hydrophobic interactions were identified between UA and HCPT molecules. In contrast, AA formed one pair of hydrophobic interactions and one hydrogen bond with HCPT, while BA established two pairs of hydrophobic interactions with HCPT. The binding energy of UA/HCPT was −4.7 kcal/mol, lower than that of AA/HCPT (−4.2 kcal/mol), OA/HCPT (−4.5 kcal/mol), and BA/HCPT (−4.2 kcal/mol), indicating stronger binding affinity for UA/HCPT.

### 3.2. Characterization

#### 3.2.1. Appearance

**Figure 2:**
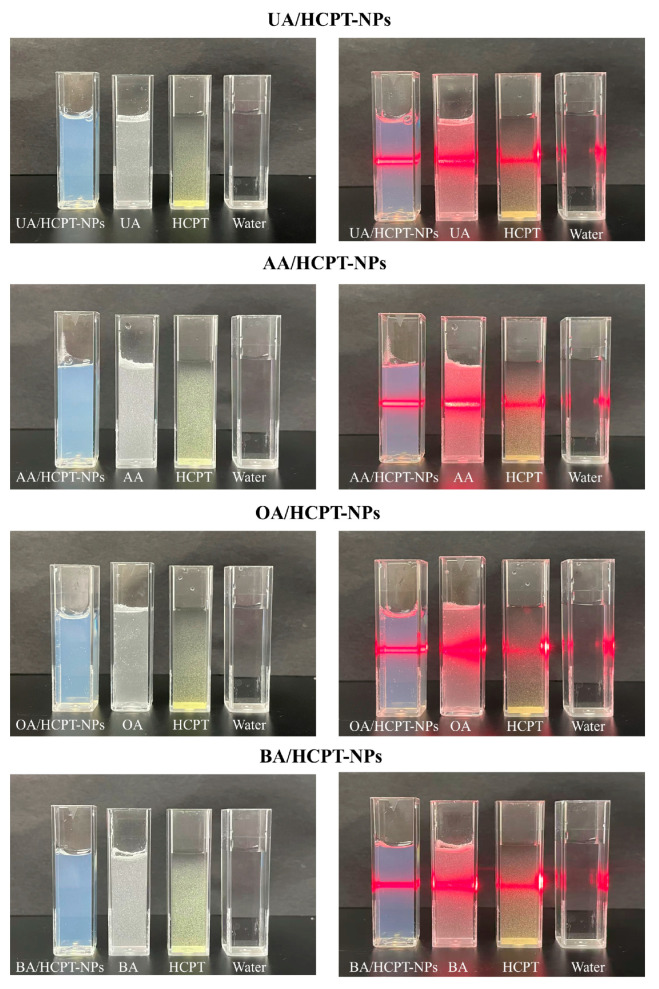
Tyndall effect in solutions of UA/HCPT-NPs, AA/HCPT-NPs, OA/HCPT-NPs, and BA/HCPT-NPs.

As presented in Figure 2, the prepared nanoparticles exhibited transparent, bluish opalescence without flocculent or granular precipitation under naked-eye observation. The nano-preparation group displayed a strong Tyndall effect with uniform optical bands under laser light, whereas the crude API dispersion group showed weak Tyndall effects, precipitate formation, and rough, short optical bands.

#### 3.2.2. Particle Size and Zeta Potential

**Figure 3:**
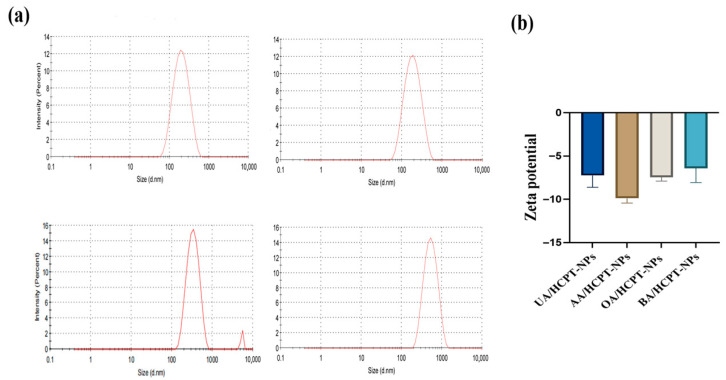
Particle size distributions (**a**) and zeta potential (**b**) of UA/HCPT-NPs, AA/HCPT-NPs, OA/HCPT-NPs, and BA/HCPT-NPs.

As shown in Figure 3, the average particle sizes of UA/HCPT-NPs, AA/HCPT-NPs, OA/HCPT-NPs, and BA/HCPT-NPs were (184.4 ± 8.6) nm, (166.6 ± 4.3) nm, (335.1 ± 1.2) nm, and (577.4 ± 54.7) nm, respectively. The zeta potential of UA/HCPT-NPs were (−7.24 ± 1.36) mV, while that of AA/HCPT-NPs, OA/HCPT-NPs, and BA/HCPT-NPs were (−9.88 ± 0.55) mV, (−7.45 ± 0.44) mV, and (−6.42 ± 1.64) mV, respectively. AA/HCPT-NPs exhibited particle size comparable to UA/HCPT-NPs, likely due to AA containing two additional hydroxyl groups that enhance intermolecular hydrogen bonding, resulting in smaller particle size.

#### 3.2.3. Stability

**Figure 4:**
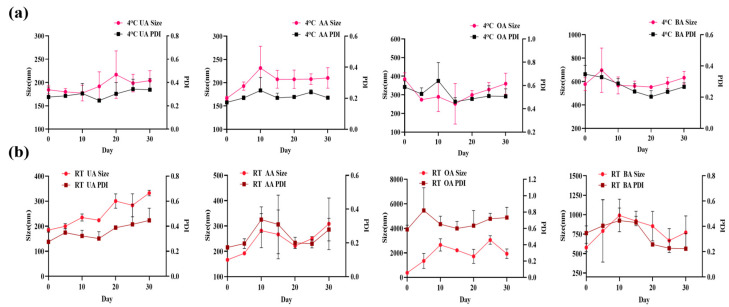
Particle size and PDI of self-assembled nanoparticles stored at 4 °C (**a**) and room temperature (**b**) for 30 days (n = 3).

Figure 4 indicated that the self-assembled nanoparticles stored at room temperature showed poor stability with significant particle size changes, while those stored at 4 °C exhibited minimal particle size changes and remained stable within 30 days.

#### 3.2.4. Scanning Electron Microscope (SEM)

**Figure 5:**
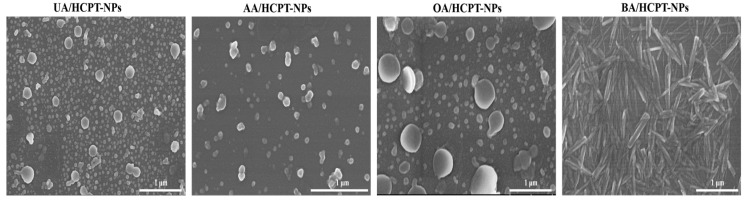
Scanning electron microscopy images (Scale bar: 1 μm) of UA/HCPT-NPs, AA/HCPT-NPs, OA/HCPT-NPs, and BA/HCPT-NPs.

SEM revealed the morphology of UA/HCPT-NPs, AA/HCPT-NPs, OA/HCPT-NPs, and BA/HCPT-NPs. As shown in Figure 5, UA/HCPT-NPs, AA/HCPT-NPs, and OA/HCPT-NPs appeared as spherical particles with sizes of ~200 nm, ~150 nm, and ~400 nm, respectively, showing uniform particle size distribution. BA/HCPT-NPs exhibited short rod-like shapes with a long-axis particle size of approximately 500 nm.

#### 3.2.5. Differential Scanning Calorimetry (DSC)

**Figure 6:**
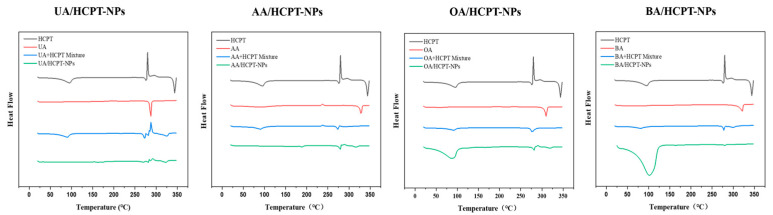
Differential scanning calorimetric thermograms of UA/HCPT-NPs, AA/HCPT-NPs, OA/HCPT-NPs, and BA/HCPT-NPs.

As illustrated in Figure 6, HCPT displayed endothermic peaks at 95.70 °C, 277.14 °C, and 344.77 °C, an exothermic peak at 300.53 °C, and a glass transition temperature of 297.10 °C. UA showed a distinct endothermic peak at 287.00 °C with a glass transition temperature of 207.15 °C. AA exhibited an exothermic peak at 237.15 °C and a prominent endothermic peak at 328.89 °C. OA had a notable endothermic peak at 310.68 °C (glass transition temperature: 66.31 °C), while BA showed an endothermic peak at 321.07 °C (glass transition temperature: 96.75 °C). The drug mixtures exhibited no significant differences in characteristic peaks compared to pure drugs. UA/HCPT-NPs displayed endothermic peaks at 161.85 °C, 173.76 °C, 269.30 °C, 281.58 °C, and 323.22 °C (glass transition temperature: 95.42 °C). AA/HCPT-NPs showed endothermic peaks at 189.26 °C, 279.33 °C, and 317.18 °C (glass transition temperature: 101.14 °C). OA/HCPT-NPs exhibited endothermic peaks at 87.28 °C, 282.35 °C, and 320.12 °C, with an exothermic peak at 290.36 °C. BA/HCPT-NPs had endothermic peaks at 101.66 °C, 162.53 °C, and 280.95 °C. These results confirm that self-assembled nanoparticles display different crystalline structures with pentacyclic triterpenoid–HCPT mixtures.

#### 3.2.6. Fourier-Transform Infrared Spectroscopy (FT-IR)

**Figure 7:**
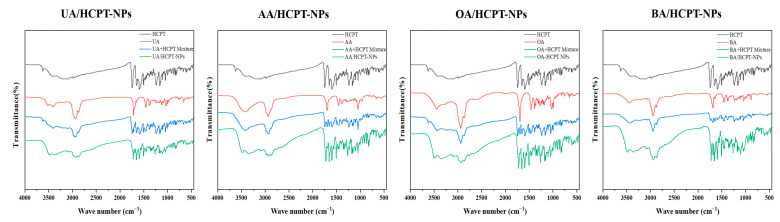
Fourier-transform infrared spectra of UA/HCPT-NPs, AA/HCPT-NPs, OA/HCPT-NPs, and BA/HCPT-NPs.

FT-IR spectra of UA, AA, OA, BA, HCPT, dual-pharmacological mixtures, and self-assembled nanoparticles are shown in Figure 7. HCPT showed characteristic peaks at 3622.86 cm^−1^ (O-H stretching) and 1747.06 cm^−1^ (carbonyl stretching in α-hydroxyl lactone ring). UA exhibited peaks at 3524.63 cm^−1^ and 3401.07 cm^−1^ (O-H stretching) and 1714.34 cm^−1^ (C=C stretching and cycloalcohol C-O stretching). AA spectrum displayed absorption bands at 3425.99 cm^−1^ (O-H stretching), 2926.59 cm^−1^ (-CH2/-CH3 stretching), 1694.95 cm^−1^ (C=O stretching), and 1049.30 cm^−1^ (C-O stretching). OA showed peaks at 3440.36 cm^−1^ (O-H stretching), 2942.42 cm^−1^ and 2864.26 cm^−1^ (-CH2 and -CH3 stretching), and 1464 cm^−1^ (C=C stretching). BA showed absorption bands at 3446.23 cm^−1^ (O-H stretching) and 1687.75 cm^−1^ (C=O stretching).

Compared with APIs, characteristic peaks of dual-pharmacological mixtures remained unchanged, indicating no significant chemical structure change in the physical mixtures. In contrast, UA/HCPT-NPs, AA/HCPT-NPs, OA/HCPT-NPs, and BA/HCPT-NPs showed pronounced changes in the 3750–2500 cm^−1^ band compared with APIs and physical mixtures, suggesting intermolecular hydrogen bonding between pentacyclic triterpenoids and HCPT during self-assembly.

### 3.3. In Vitro Antitumor Effects

#### 3.3.1. Self-Assembled Nanoparticles Enhance Antitumor Efficacy

**Figure 8:**
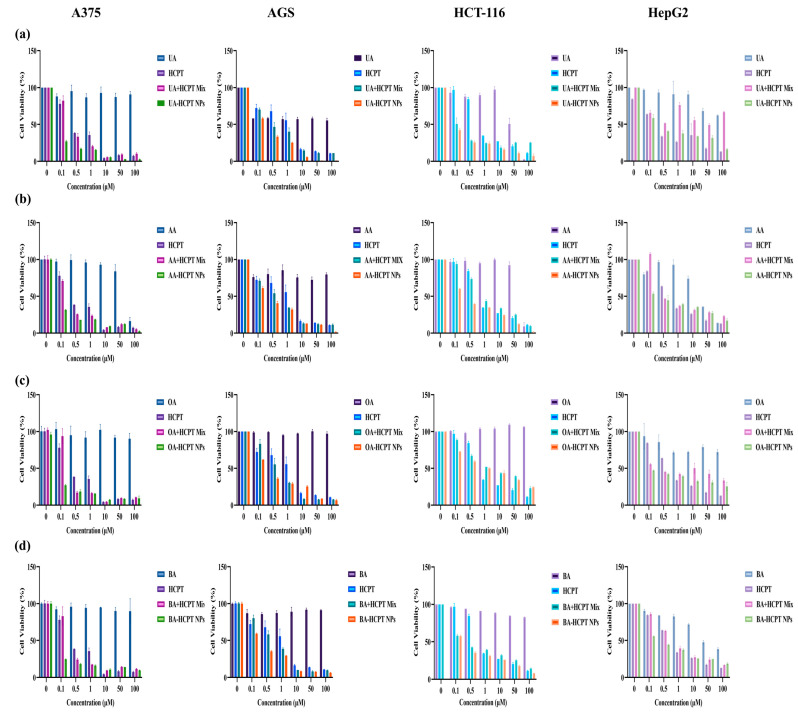
In vitro cytotoxicity of self-assembled nanoparticles against A375, AGS, HCT-116, and HepG2 cells (n = 3). (**a**) UA/HCPT-NPs, (**b**) AA/HCPT-NPs, (**c**) OA/HCPT-NPs, and (**d**) BA/HCPT-NPs co-cultured with cells at different concentrations for 48 h, evaluated by CCK-8 assay.

Figure 8 demonstrated that self-assembled nanoparticles exhibited broad-spectrum antitumor effects with concentration-dependent decreases in cell viability, indicating significant antitumor activity after 48 h. UA/HCPT-NPs showed the strongest inhibition against HepG2 cells and the weakest against AGS cells, consistent with the finding of Yan et al. [30] that oleanolic acid and ursolic acid showed dose-dependent inhibition of HepG2 cells via increased DNA fragmentation and reduced cellular activity. BA/HCPT-NPs showed slightly increased A375 cell viability at 50 μM, but viability decreased at 100 μM.

**Figure 9:**
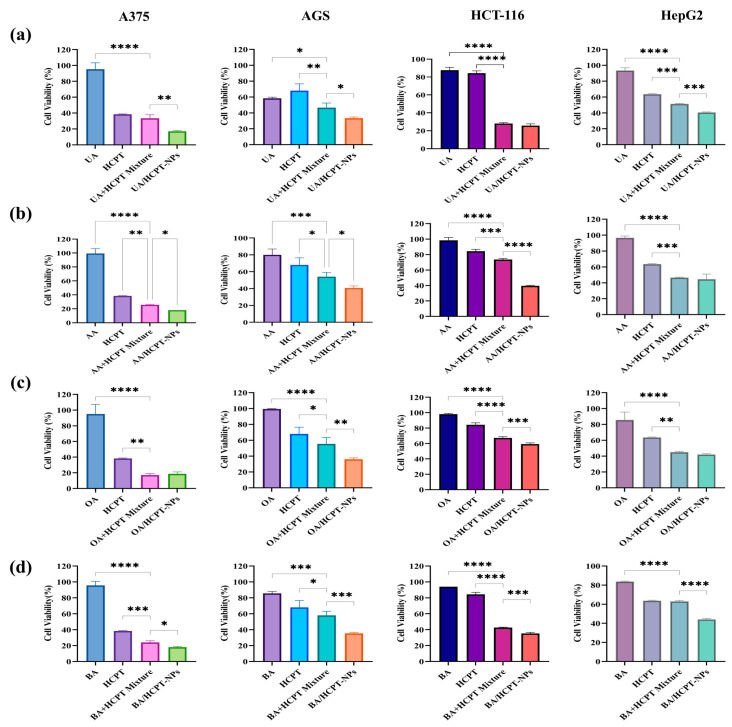
In vitro antitumor activity of self-assembled nanoparticles at 0.5 μM. A375, AGS, HCT-116, and HepG2 cells treated with (**a**) UA/HCPT-NPs, (**b**) AA/HCPT-NPs, (**c**) OA/HCPT-NPs, and (**d**) BA/HCPT-NPs for 48 h, analyzed by CCK-8 assay (n = 3). * *p* < 0.05, ** *p* < 0.01, *** *p* < 0.001, **** *p* < 0.0001.

IC_50_ values across cell lines followed the following trend: pentacyclic triterpenoids > HCPT > dual-pharmacological mixtures > self-assembled nanoparticles, see Appendix A, Table A1, Table A2, Table A3 and Table A4. Figure 9 shows that cell viability results at 0.5 μM have the same trend with the IC50 values, confirming that the combination of two drugs synergistically enhanced antitumor effects compared to monotherapies, with self-assembled nanoparticles further improving inhibition via enhanced cellular uptake.

#### 3.3.2. Enhanced Cellular Uptake of Self-Assembled Nanoparticles

**Figure 10:**
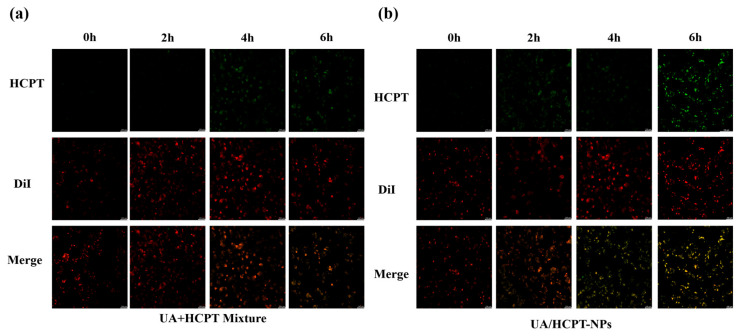
Cellular uptake of the UA + HCPT mixture and UA/HCPT-NPs observed by confocal laser scanning microscopy. DiI, a lipophilic fluorescent dye, widely applied for membrane labeling and biological tracing. CLSM images of (**a**) UA + HCPT mixture and (**b**) UA/HCPT-NPs internalized by HepG2 cells at different incubation time points (scale bar: 100 μm).

Utilizing HCPT intrinsic green fluorescence (excitation: 390 nm, emission: 435 nm) [24], cellular uptake was visualized under CLSM. As illustrated in Figure 10, HepG2 cells treated with UA/HCPT-NPs, compared with those incubated with UA + HCPT physical mixtures (5 μM HCPT equivalent), showed time-dependent increases in fluorescence intensity. UA/HCPT-NPs exhibited stronger fluorescence signals than physical mixtures, indicating superior cellular uptake, likely attributed to their smaller particle size facilitating internalization of the drug.

### 3.4. Pharmacodynamic Evaluation

#### 3.4.1. Liver Cancer of Animal Model

To validate in vivo antitumor effects, H22 tumor-bearing mice were randomized into seven groups: saline, UA (6.26 mg/kg), HCPT (5 mg/kg), UA + HCPT mixture (5 mg/kg HCPT equivalent), and UA/HCPT-NPs (2.5, 5, and 7.5 mg/kg). Tumor volumes in the mice remained relatively stable during the first two administrations. After the third administration, the saline group exhibited rapid tumor growth, while treated groups showed suppressed growth. There was a notable trend of growth in the tumor volume and a concurrent decrease in activity after the fifth administration. The 7.5 mg/kg UA/HCPT-NPs group exhibited soft stools and significant weight loss at day 4, likely attributed to high-dose toxicity, whereas other groups showed normal vital signs and feeding.

#### 3.4.2. Body Weight Changes

**Figure 11:**
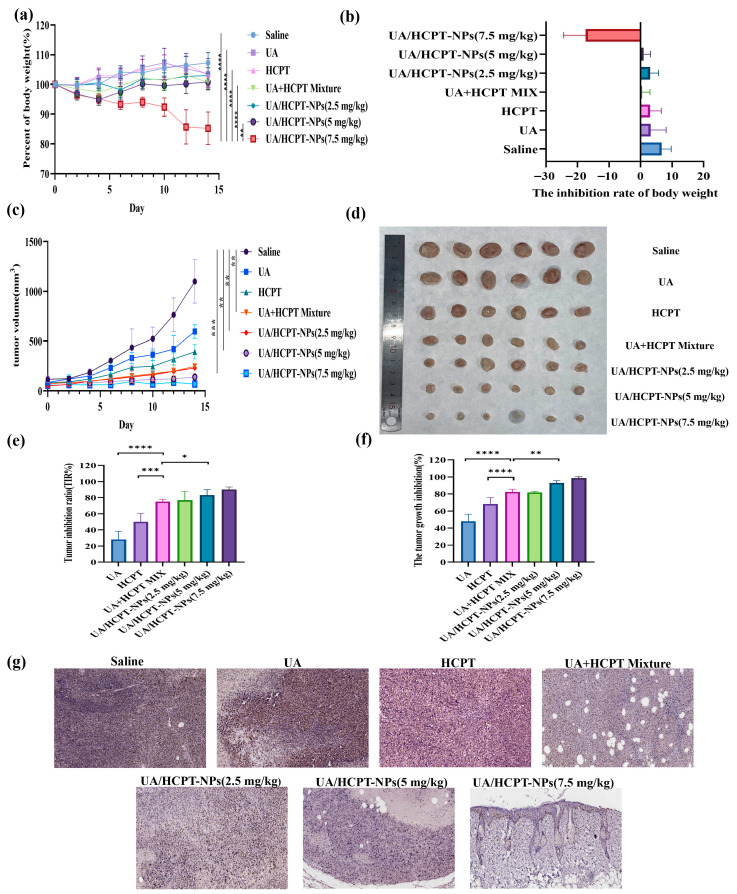
In vivo antitumor effects of UA/HCPT-NPs (n = 6). (**a**) Body weight changes during treatment. (**b**) Body weight inhibition rate. (**c**) Tumor volume dynamics. (**d**) Representative tumor tissue images. (**e**) Tumor weight inhibition rate. (**f**) Tumor growth inhibition rate. (**g**) Ki67 immunohistochemical staining of tumor tissues. * *p* < 0.05, ** *p* < 0.01, *** *p* < 0.001, **** *p* < 0.0001.

Detailed data are provided in Figure 11a. The weight change in mice is an intuitive preliminary indicator for drug safety evaluation, dynamically reflecting potential systemic adverse effects non-invasively. Recognizing that it is affected by multiple factors, we combined it with other indicators to ensure conclusion reliability [31,32]. During the treatment period, the body weight fluctuations (95–107%) in saline, UA, HCPT, UA + HCPT physical mixture, 2.5 mg/kg, and 5 mg/kg UA/HCPT-NPs indicated low toxicity. Notably, Figure 11b shows that the 7.5 mg/kg UA/HCPT-NPs group showed a 14.75% weight loss at the end of the treatment period, correlated with high-dose toxicity. No significant weight inhibition was observed in other groups (*p* > 0.05), demonstrating good tolerability and biosafety of UA/HCPT-NPs at lower doses.

#### 3.4.3. Tumor Growth Inhibition

The tumor growth inhibition rate was calculated to evaluate the efficacy of each drug group [31,32]. As shown in Figure 11c,d, the tumors of the saline group exhibited continuous and accelerated growth. UA and HCPT monotherapies reduced tumor growth rate, with HCPT showing stronger activity than UA. The UA + HCPT physical mixture significantly enhanced tumor volume inhibition compared to monotherapies. The 5 mg/kg UA/HCPT-NPs group demonstrated exceptional antitumor efficacy, with minimal tumor volume increase during treatment. The 2.5 mg/kg UA/HCPT-NPs group showed comparable antitumor efficacy within 14 days to the UA + HCPT physical mixture at half the dose, suggesting the enhanced antitumor efficacy of self-assembled nanoparticles.

As demonstrated in Figure 11e, tumor weight inhibition rates were 28.17% (UA), 50.17% (HCPT), 75.33% (UA + HCPT mixture), 76.83% (2.5 mg/kg UA/HCPT-NPs), 83.17% (5 mg/kg UA/HCPT-NPs), and 90.00% (7.5 mg/kg UA/HCPT-NPs). Compared with UA and HCPT, UA + HCPT mixture showed significantly higher inhibition (*p* < 0.0001), while 5 mg/kg UA/HCPT-NPs outperformed the mixture (*p* < 0.05), indicating promoted antitumor efficacy by the self-assembled nanoparticles. The tumor inhibition rates mirrored these trends, with UA/HCPT-NPs demonstrating dose-dependent efficacy on tumor growth (Figure 11c).

#### 3.4.4. Suppression of Tumor Cell Proliferation

Ki67 levels can reflect the proliferative activity of tumors, with higher expression level revealing stronger proliferative activity of tumor cells [33]. As illustrated in Figure 11g, Ki67 staining revealed high proliferation activity in saline and UA groups, with slightly reductions in HCPT and UA + HCPT mixture groups. All UA/HCPT-NPs dose groups exhibited significantly lower Ki67 expression, indicating potent suppression of tumor proliferation.

#### 3.4.5. Safety Evaluation

**Figure 12:**
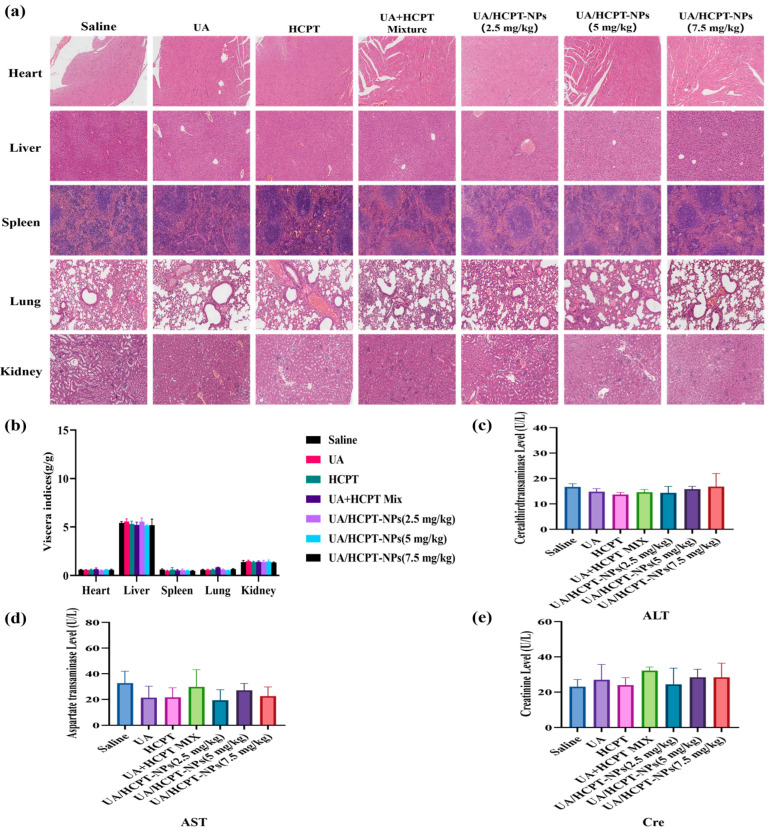
Safety evaluation of UA/HCPT-NPs (n = 6). (**a**) H&E staining of the heart, liver, spleen, lung, and kidney tissues. (**b**) Organ coefficients of mice. (**c**) ALT, (**d**) AST, and (**e**) Cre levels.

Organ coefficients, a key indicator for evaluating drug preparation safety [34], showed no significant differences across groups (Figure 12b). HE staining (Figure 12a) revealed normal organ structures in the saline group, though mild splenic lesions were observed. The UA/HCPT-NPs (7.5 mg/kg) group exhibited slight hepatic and renal lesions, likely attributed to the elevated dosage. In contrast, low-dose groups showed no overt pathological alterations, indicating that the formulation does not induce systemic toxicity and possesses acceptable safety.

Plasma levels of alanine transaminase (ALT) and aspartate aminotransferase (AST) serve as clinical markers for liver damage. ALT, predominantly located in hepatocyte cytoplasm, is a sensitive indicator of liver function damage [35], while AST, distributed in hepatocyte mitochondria, reflects severe hepatocyte damage. Plasma creatinine (Cre) levels assess renal function, with elevations indicating compromised glomerular filtration [36]. As illustrated in Figure 12c,e, ALT, AST, and Cre levels did not differ significantly across groups and remained within normal ranges, confirming that self-assembled nanoparticles do not cause severe hepatic or renal damage.

## 4. Discussion

The development of carrier-free self-assembled nanodrugs represents a promising strategy to overcome the limitations of conventional nanocarriers, such as low drug-loading, complex preparation processes, and potential carrier-induced toxicity [4]. In this study, we successfully constructed four types of self-assembled nanoparticles composed of pentacyclic triterpenoids (UA, AA, OA, BA) and 10-hydroxycamptothecin (HCPT) via an anti-solvent precipitation method. Our findings not only confirm the feasibility of co-assembly between HCPT and structurally diverse triterpenoids but also reveal the critical influence of molecular structure on the physicochemical properties and antitumor efficacy of the resulting nanodrugs.

DSC and FT-IR analyses indicated significant changes in the characteristic peaks and thermal behavior of the self-assembled NPs compared to raw materials and physical mixtures, suggesting alterations in crystalline state and the formation of hydrogen bonds during assembly. These results are consistent with previous reports on supramolecular self-assembly of natural products, where hydrogen bonding and π–π interactions play pivotal roles in stabilizing the nanostructure [4,37].

Notably, the chemical structure of the triterpenoid component significantly influenced the size, morphology, and the biological activity of the self-assembled nanoparticles [26,38]. UA/HCPT-NPs and AA/HCPT-NPs, both belonging to the ursane-type, formed spherical nanoparticles with small particle sizes. In contrast, OA/HCPT-NPs (oleanane-type) and BA/HCPT-NPs (lupane-type) exhibited larger sizes, with BA/HCPT-NPs adopting a short rod-like morphology. These differences can be attributed to variations in spatial hindrance and functional group orientation. For instance, the presence of an isopropyl group at the C-19 position in BA may introduce steric hindrance, limiting close molecular packing and leading to larger particle sizes. Such structure-dependent self-assembly behavior has been observed in other triterpenoid systems, highlighting the importance of molecular design in nanodrug development [26].

An intriguing finding from our in vitro cytotoxicity assays was the distinct and potent inhibitory profiles of different self-assembled nanoparticles against specific cancer cell lines. UA/HCPT-NPs demonstrated a pronounced cytotoxic effect against HepG2 gastric cancer cells, with an IC_50_ value significantly lower than that of the physical mixture. This suggests that the nano formulation not only enhances the delivery of the drugs but may also exert stronger cytotoxicity against HepG2 cells, which supports its potential as a targeted therapeutic agent for HepG2-derived tumors. Meanwhile, AA/HCPT-NPs exhibited strong and broad-spectrum cytotoxicity, particularly against A375 cells, AGS cells, and HCT-116 cancer cells. The superior performance of AA/HCPT-NPs could be attributed to the unique structure of AA, which possesses additional hydroxyl groups compared to UA. These groups potentially enhance hydrogen bonding within the nanoparticle, leading to a more stable and smaller nanostructure that favors cellular uptake. Similarly, OA/HCPT-NPs and BA/HCPT-NPs also showcased significant enhancement in cytotoxicity compared to their individual components or physical mixtures.

In vivo antitumor efficacy was further evaluated using H22 tumor-bearing mice. UA/HCPT-NPs exhibited the strongest tumor growth inhibition among all groups, with a clear dose-dependent effect. The tumor inhibition rates of UA/HCPT-NPs at 2.5, 5, and 7.5 mg/kg were significantly higher than those of UA, HCPT, or their physical mixture. Immunohistochemical analysis of Ki67 expression further confirmed the superior antiproliferative effect of the UA/HCPT-NPs.

Safety evaluation revealed that UA/HCPT-NPs were well tolerated at doses up to 5 mg/kg, with no significant changes in body weight, organ indices, or serum biochemical markers. However, at the highest dose (7.5 mg/kg), mild toxicity was observed, including soft stool and weight loss, along with minor pathological changes in liver and kidney tissues. This suggests that while the self-assembled nanoparticles possess a favorable safety profile, dose optimization is necessary to minimize systemic toxicity.

In summary, pentacyclic triterpenoids possess inherent self-assembly potential due to their hydrophobic skeletons and polar moieties, making them ideal for natural drug delivery systems [26,37]. The resulting nanodrugs exhibit synergistic antitumor effects, with distinct potency profiles against different cancer cell lines—such as the marked efficacy of UA/HCPT-NPs against HepG2 cells and the remarkably broad-spectrum cytotoxicity of AA/HCPT-NPs. These effects are mediated by enhanced cellular uptake and synergistic drug interactions. These findings provide a foundation for the development of multidrug self-assembled nanosystems for combination and potentially personalized cancer therapy.

This study demonstrates the combined antitumor activity of dual drugs and confirms their general self-assembly phenomenon. Future studies should focus on in vivo targeting strategies, long-term toxicity, and the mechanisms underlying the cell-type-specific synergistic effects of these novel nanomedicines.

## Data Availability

The datasets used and analyzed during the current study are available from the corresponding author on request.

## Abbreviations

The following abbreviations are used in this manuscript:

HCPT	10-hydroxycamptothecin
UA	Ursolic acid
AA	Asiatic acid
OA	Oleanic acid
BA	Betulinic acid
2D	Two-dimensional
3D	Three-dimensional
APIs	Active pharmaceutical ingredient
CCK-8	Cell Counting Kit-8
CLSM	Confocal laser scanning microscopy
DMSO	Dimethyl sulfoxide
P188	Poloxamer 188
SEM	Scanning electron microscope
PDI	Polymer dispersity index
DSC	Differential scanning calorimetry
FT-IR	Fourier-transform infrared spectroscopy
IC_50_	Half-maximal inhibitory concentration
DiI	1,1′-dioctadecyl-3,3,3′,3′-tetramethylindocarbocyanine perchlorate
IRBW	Inhibition rate of body weight
TWIR	Tumor weight inhibition rate
TGI	Tumor growth inhibition
Cre	Creatinine
ALT	Alanine transaminase
AST	Aspartate aminotransferase

## Appendix A

**Table A1:** IC50 values of UA, HCPT, UA + HCPT physical mixture, and UA/HCPT-NPs against different tumor cells (μM).

	UA	HCPT	UA + HCPT Mixture	UA/HCPT-NPs	Title 1	Title 2	Title 3
A375	0.104	0.305	0.2363	4.538 × 10^−10^	entry 1	data	data
AGS	115.100	1.923	0.450	0.116	entry 2	data	data
HCT-116	50.280	0.699	0.164	4.848 × 10^−12^			
HepG2	163.000	0.214	0.071	8.043 × 10^−16^			

**Table A2:** IC50 values of AA, HCPT, AA + HCPT physical mixture, and AA/HCPT-NPs against different tumor cells (μM).

	AA	HCPT	AA + HCPT Mixture	AA/HCPT-NPs		UA	HCPT	UA + HCPT Mixture	UA/HCPT-NPs	Title 1	Title 2	Title 3
A375	1795	0.3050	0.1848	4.954 × 10^−9^	A375	0.104	0.305	0.2363	4.538 × 10^−10^	entry 1	data	data
AGS	9.184	1.923	0.6872	0.2491	AGS	115.100	1.923	0.450	0.116	entry 2	data	data
HCT-116	98.60	0.6992	2.303× 10^−5^	2.749 × 10^−42^	HCT-116	50.280	0.699	0.164	4.848 × 10^−12^			
HepG2	51.15	0.2141	3.049 × 10^−5^	1.010 × 10^−16^	HepG2	163.000	0.214	0.071	8.043 × 10^−16^			

**Table A3:** IC50 values of OA, HCPT, OA + HCPT physical mixture, and OA/HCPT-NPs against different tumor cells (μM).

	OA	HCPT	OA + HCPT Mixture	OA/HCPT-NPs		UA	HCPT	UA + HCPT Mixture	UA/HCPT-NPs	Title 1	Title 2	Title 3
A375	22.430	0.305	0.243	7.961 × 10^−8^	A375	0.104	0.305	0.2363	4.538 × 10^−10^	entry 1	data	data
AGS	-	1.923	0.608	3.202 × 10^−13^	AGS	115.100	1.923	0.450	0.116	entry 2	data	data
HCT-116	10.080	0.699	4.908 × 10^−10^	2.154 × 10^−40^	HCT-116	50.280	0.699	0.164	4.848 × 10^−12^			
HepG2	0.5120	0.214	0.054	1.139 × 10^−11^	HepG2	163.000	0.214	0.071	8.043 × 10^−16^			

**Table A4:** IC50 values of BA, HCPT, BA + HCPT physical mixture, and BA/HCPT-NPs against different tumor cells (μM).

	BA	HCPT	BA + HCPT Mixture	BA/HCPT-NPs		UA	HCPT	UA + HCPT Mixture	UA/HCPT-NPs	Title 1	Title 2	Title 3
A375	45.890	0.305	0.235	2.606 × 10^−7^	A375	0.104	0.305	0.2363	4.538 × 10^−10^	entry 1	data	data
AGS	10.680	1.923	0.746	0.516	AGS	115.100	1.923	0.450	0.116	entry 2	data	data
HCT-116	24.040	0.699	0.039	5.575 × 10^−16^	HCT-116	50.280	0.699	0.164	4.848 × 10^−12^			
HepG2	546.600	0.214	0.060	6.344 × 10^−11^	HepG2	163.000	0.214	0.071	8.043 × 10^−16^			
